# Bis{2-[2-(dimethyl­amino)ethyl­imino­meth­yl]-4,6-disulfanylphenolato}cobalt(II) monohydrate

**DOI:** 10.1107/S1600536809033650

**Published:** 2009-08-29

**Authors:** Ying-Jie Cai, Jin Li, Peng Huang, Qing-Fu Zeng

**Affiliations:** a Engineering Research Center for Clean Production of Textile Dyeing and Printing, Ministry of Education, Wuhan 430073, People’s Republic of China

## Abstract

In the title hydrated complex, [Co(C_11_H_15_N_2_OS_2_)_2_]·H_2_O, the Co^II^ atom (site symmetry 2) is coordinated by two *O*,*N*,*N*′-tridentate Schiff base ligands, resulting in a very distorted *cis*-CoO_2_N_4_ octa­hedral geometry for the metal ion. In the crystal, the water mol­ecule (O-atom site symmetry 2) inter­acts with nearby complex mol­ecules by way of bifurcated O—H⋯(O,S) hydrogen bonds.

## Related literature

For a related compound and background, see: Li *et al.* (2009 ▶). For reference structural data, see: Allen *et al.* (1987 ▶).
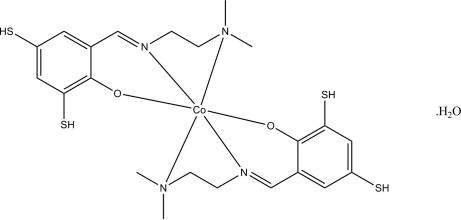


## Experimental

### 

#### Crystal data

[Co(C_11_H_15_N_2_OS_2_)_2_]·H_2_O
*M* *_r_* = 587.69
Orthorhombic, 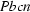

*a* = 12.3755 (15) Å
*b* = 9.2485 (15) Å
*c* = 22.827 (3) Å
*V* = 2612.7 (6) Å^3^
*Z* = 4
Mo *K*α radiation
μ = 1.01 mm^−1^
*T* = 296 K
0.30 × 0.25 × 0.25 mm

#### Data collection

Enraf–Nonius CAD-4 diffractometer
Absorption correction: ψ scan (North *et al.*, 1968 ▶) *T*_min_ = 0.752, *T*_max_ = 0.787
13847 measured reflections
2548 independent reflections
2039 reflections with *I* > 2σ(*I*)
*R*_int_ = 0.031
200 standard reflections every 3 reflections intensity decay: 1%

#### Refinement

*R*[*F* ^2^ > 2σ(*F* ^2^)] = 0.054
*wR*(*F* ^2^) = 0.186
*S* = 1.06
2548 reflections
160 parameters
1 restraint
H atoms treated by a mixture of independent and constrained refinement
Δρ_max_ = 0.84 e Å^−3^
Δρ_min_ = −0.96 e Å^−3^

### 

Data collection: *CAD-4 Software* (Enraf–Nonius, 1989 ▶); cell refinement: *CAD-4 Software*; data reduction: *XCAD4* (Harms & Wocadlo, 1995 ▶); program(s) used to solve structure: *SHELXS97* (Sheldrick, 2008 ▶); program(s) used to refine structure: *SHELXL97* (Sheldrick, 2008 ▶); molecular graphics: *SHELXTL* (Sheldrick, 2008 ▶); software used to prepare material for publication: *SHELXTL*.

## Supplementary Material

Crystal structure: contains datablocks global, I. DOI: 10.1107/S1600536809033650/hb5056sup1.cif

Structure factors: contains datablocks I. DOI: 10.1107/S1600536809033650/hb5056Isup2.hkl

Additional supplementary materials:  crystallographic information; 3D view; checkCIF report

## Figures and Tables

**Table 1:** Selected bond lengths (Å)

Co1—O1	2.089 (2)
Co1—N2	2.241 (3)
Co1—N1	2.456 (3)

**Table 2:** Hydrogen-bond geometry (Å, °)

*D*—H⋯*A*	*D*—H	H⋯*A*	*D*⋯*A*	*D*—H⋯*A*
O2—H2*A*⋯S2^i^	0.832 (10)	2.95 (2)	3.7001 (17)	151 (4)
O2—H2*A*⋯O1^i^	0.832 (10)	2.25 (3)	2.928 (5)	139 (4)

Symmetry code: (i) 
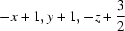
.

**Fig. 1.**
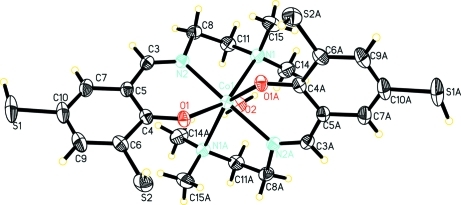
The molecular structure of (I) showing 30% probability displacement ellipsoids. Atoms with the suffix A are generated by the symmetry operation (1–x, y, 3/2–z).

## supplementary crystallographic information

### Comment

As part of our onging studies of this family of compounds (Li *et al.*,  2009), we report here the crystal structure of the title compound, (I). In (I), all bond lengths are within normal ranges (Allen *et al.*, 1987) (Fig. 1). The Co(II) is six-coordinated in a distorted octhaedral coordination by two N,N,O-tridentate Schiff base ligands.

### Experimental

A mixture of 2-hydroxy-3,5-dimercaptobenzaldehyde (372 mg, 2 mmol), *N*,*N*-dimethylethane-1,2-diamine (176 mg, 2 mmol) and CoCl_2_.6H_2_O (1 mmol, 238 mg) in methanol (10 ml) was stirred for 1 h. After keeping the filtrate in air for 7 d, red blocks of (I) were formed.

### Refinement

The water H atom was located in a difference map and its position was freely refined. All other H atoms were positioned geometrically (C—H = 0.93–0.97Å, S—H = 1.2Å) and refined as riding, with *U*_iso_(H) = 1.2*U*_eq_(carrier) or 1.5*U*_eq_(methyl C).

### Crystal data

**Table:**

[Co(C_11_H_15_N_2_OS_2_)_2_]·H_2_O	*F*(000) = 1228
*M**_r_* = 587.69	*D*_x_ = 1.494 Mg m^−^^3^
Orthorhombic, *P**b**c**n*	Mo *K*α radiation, λ = 0.71073 Å
Hall symbol: -P 2n 2ab	Cell parameters from 25 reflections
*a* = 12.3755 (15) Å	θ = 9–12°
*b* = 9.2485 (15) Å	µ = 1.01 mm^−^^1^
*c* = 22.827 (3) Å	*T* = 296 K
*V* = 2612.7 (6) Å^3^	Block, red
*Z* = 4	0.30 × 0.25 × 0.25 mm

### Data collection

**Table:**

Enraf–Nonius CAD-4 diffractometer	2039 reflections with *I* > 2σ(*I*)
Radiation source: fine-focus sealed tube	*R*_int_ = 0.031
graphite	θ_max_ = 26.0°, θ_min_ = 2.4°
ω/2θ scans	*h* = −15→15
Absorption correction: ψ scan (North *et al.*, 1968)	*k* = −11→6
*T*_min_ = 0.752, *T*_max_ = 0.787	*l* = −28→27
13847 measured reflections	200 standard reflections every 3 reflections
2548 independent reflections	intensity decay: 1%

### Refinement

**Table:**

Refinement on *F*^2^	Primary atom site location: structure-invariant direct methods
Least-squares matrix: full	Secondary atom site location: difference Fourier map
*R*[*F*^2^ > 2σ(*F*^2^)] = 0.054	Hydrogen site location: inferred from neighbouring sites
*wR*(*F*^2^) = 0.186	H atoms treated by a mixture of independent and constrained refinement
*S* = 1.06	*w* = 1/[σ^2^(*F*_o_^2^) + (0.1201*P*)^2^ + 2.1692*P*] where *P* = (*F*_o_^2^ + 2*F*_c_^2^)/3
2548 reflections	(Δ/σ)_max_ = 0.001
160 parameters	Δρ_max_ = 0.84 e Å^−^^3^
1 restraint	Δρ_min_ = −0.96 e Å^−^^3^

### Special details

**Table:**

Geometry. All e.s.d.'s (except the e.s.d. in the dihedral angle between two l.s. planes) are estimated using the full covariance matrix. The cell e.s.d.'s are taken into account individually in the estimation of e.s.d.'s in distances, angles and torsion angles; correlations between e.s.d.'s in cell parameters are only used when they are defined by crystal symmetry. An approximate (isotropic) treatment of cell e.s.d.'s is used for estimating e.s.d.'s involving l.s. planes.
Refinement. Refinement of *F*^2^ against ALL reflections. The weighted *R*-factor *wR* and goodness of fit *S* are based on *F*^2^, conventional *R*-factors *R* are based on *F*, with *F* set to zero for negative *F*^2^. The threshold expression of *F*^2^ > σ(*F*^2^) is used only for calculating *R*-factors(gt) *etc*. and is not relevant to the choice of reflections for refinement. *R*-factors based on *F*^2^ are statistically about twice as large as those based on *F*, and *R*- factors based on ALL data will be even larger.

### Fractional atomic coordinates and isotropic or equivalent isotropic displacement parameters (Å^2^)

**Table:**

	*x*	*y*	*z*	*U*_iso_*/*U*_eq_	
C3	0.2776 (3)	0.1848 (3)	0.68222 (16)	0.0417 (8)	
H3	0.2082	0.2207	0.6768	0.050*	
C4	0.4167 (3)	0.0107 (3)	0.64302 (14)	0.0380 (7)	
C5	0.3168 (3)	0.0854 (4)	0.63802 (14)	0.0395 (7)	
C6	0.4439 (3)	−0.0791 (4)	0.59437 (15)	0.0467 (9)	
C7	0.2502 (3)	0.0634 (4)	0.58895 (17)	0.0524 (10)	
H7	0.1846	0.1120	0.5866	0.063*	
C8	0.2678 (3)	0.3286 (4)	0.76616 (19)	0.0507 (9)	
H8A	0.2245	0.2739	0.7938	0.061*	
H8B	0.2195	0.3881	0.7429	0.061*	
C9	0.3788 (4)	−0.0974 (4)	0.54666 (16)	0.0588 (11)	
H9	0.4004	−0.1560	0.5157	0.071*	
C10	0.2796 (4)	−0.0272 (5)	0.54503 (18)	0.0623 (12)	
C11	0.3458 (3)	0.4236 (4)	0.79892 (18)	0.0514 (9)	
H11A	0.3791	0.4909	0.7718	0.062*	
H11B	0.3066	0.4795	0.8279	0.062*	
C14	0.5090 (4)	0.4381 (5)	0.8533 (2)	0.0673 (13)	
H14A	0.5339	0.5032	0.8234	0.101*	
H14B	0.5692	0.3847	0.8686	0.101*	
H14C	0.4757	0.4922	0.8842	0.101*	
C15	0.3836 (4)	0.2501 (5)	0.87496 (19)	0.0651 (12)	
H15A	0.3375	0.3091	0.8989	0.098*	
H15B	0.4402	0.2100	0.8987	0.098*	
H15C	0.3421	0.1732	0.8579	0.098*	
Co1	0.5000	0.18183 (7)	0.7500	0.0390 (3)	
H2A	0.502 (3)	0.789 (4)	0.7815 (11)	0.059 (14)*	
N1	0.4310 (2)	0.3386 (3)	0.82838 (13)	0.0431 (7)	
N2	0.3272 (2)	0.2283 (3)	0.72746 (13)	0.0401 (6)	
O1	0.48006 (19)	0.0189 (3)	0.68755 (11)	0.0412 (6)	
O2	0.5000	0.7436 (5)	0.7500	0.0605 (11)*	
S1	0.19385 (15)	−0.05824 (19)	0.48627 (6)	0.0990 (6)	
H1	0.1107	0.0061	0.4937	0.148*	
S2	0.56823 (10)	−0.16647 (13)	0.59645 (5)	0.0678 (4)	
H2	0.5567	−0.2860	0.6159	0.102*	

### Atomic displacement parameters (Å^2^)

**Table:**

	*U*^11^	*U*^22^	*U*^33^	*U*^12^	*U*^13^	*U*^23^
C3	0.0367 (18)	0.0393 (18)	0.049 (2)	−0.0001 (13)	−0.0046 (14)	0.0043 (14)
C4	0.0496 (19)	0.0308 (15)	0.0336 (16)	−0.0073 (14)	−0.0017 (14)	0.0007 (12)
C5	0.0439 (18)	0.0357 (16)	0.0391 (17)	−0.0069 (14)	−0.0053 (14)	0.0024 (13)
C6	0.062 (2)	0.0407 (18)	0.0372 (17)	−0.0006 (16)	0.0042 (16)	−0.0029 (14)
C7	0.055 (2)	0.054 (2)	0.048 (2)	−0.0105 (18)	−0.0152 (17)	0.0112 (16)
C8	0.042 (2)	0.055 (2)	0.056 (2)	0.0115 (16)	0.0015 (17)	−0.0060 (17)
C9	0.086 (3)	0.053 (2)	0.0371 (19)	−0.012 (2)	−0.0050 (19)	−0.0067 (16)
C10	0.085 (3)	0.061 (2)	0.041 (2)	−0.021 (2)	−0.022 (2)	0.0033 (18)
C11	0.057 (2)	0.0418 (19)	0.055 (2)	0.0080 (17)	0.0037 (18)	−0.0054 (16)
C14	0.056 (3)	0.068 (3)	0.078 (3)	−0.004 (2)	0.003 (2)	−0.028 (2)
C15	0.076 (3)	0.066 (3)	0.054 (2)	0.011 (2)	0.018 (2)	0.004 (2)
Co1	0.0383 (4)	0.0381 (4)	0.0406 (4)	0.000	−0.0028 (2)	0.000
N1	0.0433 (16)	0.0398 (15)	0.0462 (16)	0.0012 (12)	0.0001 (13)	−0.0065 (12)
N2	0.0376 (15)	0.0375 (14)	0.0452 (16)	0.0029 (11)	−0.0013 (12)	−0.0018 (13)
O1	0.0498 (13)	0.0367 (12)	0.0372 (12)	0.0030 (10)	−0.0080 (10)	−0.0059 (10)
S1	0.1182 (13)	0.1170 (13)	0.0616 (8)	−0.0154 (9)	−0.0498 (8)	−0.0119 (7)
S2	0.0758 (8)	0.0646 (7)	0.0629 (7)	0.0195 (6)	0.0044 (5)	−0.0196 (5)

### Geometric parameters (Å, °)

**Table:**

C3—N2	1.267 (5)	C11—H11A	0.9700
C3—C5	1.449 (5)	C11—H11B	0.9700
C3—H3	0.9300	C14—N1	1.449 (5)
C4—O1	1.286 (4)	C14—H14A	0.9600
C4—C5	1.421 (5)	C14—H14B	0.9600
C4—C6	1.427 (5)	C14—H14C	0.9600
C5—C7	1.406 (5)	C15—N1	1.464 (5)
C6—C9	1.366 (5)	C15—H15A	0.9600
C6—S2	1.738 (4)	C15—H15B	0.9600
C7—C10	1.356 (6)	C15—H15C	0.9600
C7—H7	0.9300	Co1—O1	2.089 (2)
C8—N2	1.477 (5)	Co1—O1^i^	2.089 (2)
C8—C11	1.505 (6)	Co1—N2^i^	2.241 (3)
C8—H8A	0.9700	Co1—N2	2.241 (3)
C8—H8B	0.9700	Co1—N1	2.456 (3)
C9—C10	1.389 (7)	Co1—N1^i^	2.456 (3)
C9—H9	0.9300	O2—H2A	0.832 (10)
C10—S1	1.735 (4)	S1—H1	1.2000
C11—N1	1.478 (5)	S2—H2	1.2000
			
N2—C3—C5	127.4 (3)	H11A—C11—H11B	107.9
N2—C3—H3	116.3	N1—C14—H14A	109.5
C5—C3—H3	116.3	N1—C14—H14B	109.5
O1—C4—C5	124.4 (3)	H14A—C14—H14B	109.5
O1—C4—C6	120.4 (3)	N1—C14—H14C	109.5
C5—C4—C6	115.2 (3)	H14A—C14—H14C	109.5
C7—C5—C4	120.2 (3)	H14B—C14—H14C	109.5
C7—C5—C3	116.8 (3)	N1—C15—H15A	109.5
C4—C5—C3	123.0 (3)	N1—C15—H15B	109.5
C9—C6—C4	123.6 (4)	H15A—C15—H15B	109.5
C9—C6—S2	119.1 (3)	N1—C15—H15C	109.5
C4—C6—S2	117.3 (3)	H15A—C15—H15C	109.5
C10—C7—C5	121.4 (4)	H15B—C15—H15C	109.5
C10—C7—H7	119.3	O1—Co1—O1^i^	87.65 (14)
C5—C7—H7	119.3	O1—Co1—N2^i^	114.06 (10)
N2—C8—C11	110.2 (3)	O1^i^—Co1—N2^i^	82.47 (10)
N2—C8—H8A	109.6	O1—Co1—N2	82.48 (10)
C11—C8—H8A	109.6	O1^i^—Co1—N2	114.06 (10)
N2—C8—H8B	109.6	N2^i^—Co1—N2	157.88 (15)
C11—C8—H8B	109.6	N1—Co1—O1	151.87 (12)
H8A—C8—H8B	108.1	N1—Co1—N2	73.88 (12)
C6—C9—C10	119.0 (4)	N1—Co1—N1^i^	107.64 (12)
C6—C9—H9	120.5	C14—N1—C15	109.7 (4)
C10—C9—H9	120.5	C14—N1—C11	108.4 (3)
C7—C10—C9	120.4 (4)	C15—N1—C11	110.0 (3)
C7—C10—S1	120.7 (4)	C3—N2—C8	116.5 (3)
C9—C10—S1	118.9 (3)	C3—N2—Co1	126.0 (2)
N1—C11—C8	111.9 (3)	C8—N2—Co1	117.3 (2)
N1—C11—H11A	109.2	C4—O1—Co1	130.8 (2)
C8—C11—H11A	109.2	C10—S1—H1	109.5
N1—C11—H11B	109.2	C6—S2—H2	109.5
C8—C11—H11B	109.2		

Symmetry codes: (i) −*x*+1, *y*, −*z*+3/2.

### Hydrogen-bond geometry (Å, °)

**Table:**

*D*—H···*A*	*D*—H	H···*A*	*D*···*A*	*D*—H···*A*
O2—H2A···S2^ii^	0.83 (1)	2.95 (2)	3.7001 (17)	151 (4)
O2—H2A···O1^ii^	0.83 (1)	2.25 (3)	2.928 (5)	139 (4)

Symmetry codes: (ii) −*x*+1, *y*+1, −*z*+3/2.
